# Voluntary Hospital Finance

**Published:** 1917-04-21

**Authors:** 


### VOLUNTARY HOSPITAL FINANCE.

### I. Some Reflections and a Foreword.

It is a remarkable fact, but it is a fact, that despite the war the voluntary hospitals have weathered their financial difficulties, especially where the management has been of the best, to an extent which justifies those who believe in the voluntary system to thank God and take courage. In the five years for which the latest figures are available voluntary hospital expenditure in the United Kingdom has increased by £486,000, a fact which, taken with the expenditure for the last of the years included, justifies the expectation that the average expenditure of our voluntary hospitals will continue to increase at the rate of fully £100,000 a year. The increase in the ordinary income of these hospitals during the five years amounted to £350,000—that is to say, it was on an average £27,000 a year less than the increase in expenditure. This difference has been met by the yield from legacies, which has been on the whole excellent. The yield from legacies in 1914 in London was greater than in 1913, and although this increase was not maintained in the provincial, Scottish, and Irish hospitals in 1914 compared with 1913, still legacies for the five years are, on the whole, very encouraging. During the five years ending 1914 the total surplus income for the five years amounted to £1,617,000, that is an average surplus of £323,400. It is interesting in this connection to observe that the special donations fell off materially during each of the five years.

This is not surprising, as donations are probably the most fluctuating of all channels of revenue to voluntary hospitals, and they are dependent largely upon the amount of work put into appeals, and the amount of money expended upon such special efforts each year. There is nothing which falls off so readily as the casual donation, and the wise have followed a plan which often proves fruitful, by classifying the donations actually received into donations and annual donations, that often yields good direct and indirect results. Direct in the sense that a polite letter addressed to the donor about the time the last donation was received will generally bring a renewal of the gift. Indirect, because Anno Domini affects the voluntary supporters of hospitals, as it affects everybody else. As the giver grows older, friends die, relations grow old or are scattered, and the tendency each year is for the elders who survive to find themselves more and more isolated, because the making of friends is an art which weakens to the vanishing point after the attainment of a certain age. Consequently, the knowledgeable secretary, who keeps in touch with all his supporters and makes friends of them, is rejoiced over when the postman brings a letter from him asking for the renewal of the (annual) donation; and when the time for the will to be made arrives, the particular hospital which is blessed with such an officer is frequently entered on the list of legacies. Looking at the matter all round, every able administrator, responsible for the finances of a well-conducted voluntary hospital, has the best of reasons for exhibiting complete confidence and freedom from anxiety as to the future yield of revenue in support of the work of his hospital. The very fact that the special donations may have fallen off steadily during the years of war is a matter fully anticipated by the wise, who are quite willing to stand aside and let the war have its share of this casual revenue, knowing that, directly the war is over, if the financial machinery of the hospital is right, the yield from special donations can be restored by increased vigour, with encouraging fruitfulness.

We have been dealing with the figures of those hospitals which wisely have a financial department, ably officered, and so organised as to economise expenditure and appeals by only scattering the latter through approved fields of possibility, that is to persons who it is known have the means, if they can be brought to exhibit the will, to give, and give liberally, to hospitals. Further, that no appeal shall be issued at any season of the year when common-sense and experience combine to prove that it is but a waste of paper and postage to send it out. We know by experience—and our letter-box proves this to us with increasing force every year—that as the number of institutions making appeals increases, the amount of waste in paper and postage stamps, due to ignorance, absence of training, and of fulness of knowledge, has grown to proportions which show something wrong with the management of numerous charities. Fortunately, the majority of voluntary hospitals manage their business on wiser lines, or it would not have been possible for the revenue of our hospitals to show a total surplus of over £300,000 during each of the last five years. Good management has resulted in the actual revenues of the voluntary hospitals, even in wartime, being surprisingly good. We believe that those voluntary hospitals which have had the wisdom to devote as many beds as possible to the reception of wounded warriors, and to take payment from the Government for them, will be found, deservedly, amongst the most popular and best supported by the public at the end of the war. The London hospitals should never forget that they have now behind them the invaluable support in money and prestige which is extended to them through the work of King Edward's Hospital Fund, its awards, and the annual Statistical Reports, which that Fund generously distributes to all hospital people, and, indeed, to all who have reason to ask for a copy of them.

The question has arisen as to whether or not it is desirable during wartime to have an annual dinner. To that question we would add another. Why should a hospital lay itself out to appeal, once in two, three, or five years, with full knowledge for the funds it needs to pay its way? We link these two propositions together, seeing that in the case of a biennial or quinquennial appeal the amount to be raised always includes a substantial sum to start with—namely, that from the faithful supporters who have during successive appeals invariably put their names down to give £x during each of the two or five years for which the latest appeal is made. The annual dinner list from time immemorial has usually included the names of departments, institutions, and large givers, who have contributed to the dinner fund, probably without intermission, or nearly so, since they first became interested in the hospital or charity on whose behalf the festival is held. Years ago we made an analysis of the actual sums which would have come to the hospital without a dinner at all, and found them to be so considerable that when the expense of the dinner was saved, and a fair debtor and creditor account was made out, on business considerations alone the dinner in most instances was poor or even bad business, because the sum represented by the difference between the regular contributors to it, who could be got without a dinner, and the total receipts after deducting the expenses when the new givers were added, was a sum readily compassable by a little special energy and the ordinary appeal work of the institution. Our inquiries and investigations convince us, too, that the same may be said with practically equal force and truth of biennial, triennial, and quinquennial appeals. A great hospital needs continuous vitality in the conduct of its financial affairs. This vitality is best secured by the expenditure of continuous energy to the fullest extent by those responsible for the financial management of the institution. On this system it should be possible to secure that the yield of revenue shall exceed the outlay on expenditure, whenever it may be thought desirable to produce such a result.

The time has gone by when any man of experience in hospital finance will suffer his institution to run the risk of disaster by putting a statement in his appeal to the effect that if such and such a sum is not forthcoming beds will be closed. The British public knows that the closing of beds by any hospital means extravagant and faulty management. There is no way out of this conclusion, that the larger givers, the wisest givers, and the most devoted and interested givers to our voluntary hospitals, too, have grasped this fact, and have been acting upon it for at least two decades of years, when apportioning their contributions to the hospitals they may favour.

(*To be continued.*)

